# Editorial: On the Inflammatory Cascade-From Bacteria Through the Epithelium to the Connective Tissue

**DOI:** 10.3389/fcimb.2022.936833

**Published:** 2022-06-10

**Authors:** Farah Asa’ad, Young-Dan Cho, Lena Larsson

**Affiliations:** ^1^ Department of Biomaterials, Institute of Clinical Sciences, The Sahlgrenska Academy at University of Gothenburg, Göteborg, Sweden; ^2^ Department of Oral Biochemistry, Institute of Odontology, The Sahlgrenska Academy at University of Gothenburg, Göteborg, Sweden; ^3^ Department of Periodontology, School of Dentistry and Dental Research Institute, Seoul National University and Seoul National University Dental Hospital, Seoul, South Korea; ^4^ Department of Periodontology, Institute of Odontology, The Sahlgrenska Academy at University of Gothenburg, Göteborg, Sweden

**Keywords:** inflammation, bacteria, epithelium, cell signaling, immune response

The mucosa maintains a functional barrier protecting against chemical, physical, and biological insults. The innate immune response is the first line of defense against pathogens. When the innate immune response detects a danger signal, such as bacteria, tumorigenesis and tissue damage, through the Pathogen Recognition Receptors (PRRs), it initiates an inflammatory response. If this response is not regulated, it can lead to an uncontrolled inflammation, septic shock, cancer or autoimmune diseases. Several mechanisms, including epigenetic changes, have been suggested to influence the downstream signaling from epithelial cells to inflammatory cells in the connective tissue thereby further regulating the inflammatory processes not only in the innate immune response but also the adapted immune response. Chronic inflammation and/or abrupt microbial imbalance (dysbiosis) dynamically alter cellular and molecular events, leading to the development of disease such as inflammatory bowel disease, irritable bowel syndrome, celiac disease, colitis, and periodontitis among other diseases.

This Research Topic comprises new & various research on how bacteria influence the inflammatory cascade and the reaction of different tissues to pathogenic bacteria.

In a clinical case presented by Johansson et al., the tissue response to bone-anchored hearing implant & the occurrence of bacterial infection was assessed by multiscale and multimodal analysis of an implant removed due to pain two-years post-implantation, for no apparent reason. Their comprehensive analysis confirmed polymicrobial colonization in the peri-abutment area and on the implant, including that with *Staphylococcus aureus* and *Staphylococcus epidermidis.* As such, these results might indicate that despite implant osseointegration and the absence of macroscopic signs of infection, chronic pain related to the bone-anchoring hearing implants could be associated with a chronic bacterial infection and raised inflammatory response.

In an *in vitro* study, Ueda et al. gave an in-depth mechanistic insight on how systemic infections, such as sepsis and meningitis, could occur due to invasion of *Aeromonas sobria*, a pathogenic bacterium that is responsible for food-borne illness, from the intestinal epithelium. Using intestinal cultured cells (T84 cells), the authors observed that serine protease produced by *Aeromonas sobria* caused destruction of some of the protein components, such as ZO proteins and claudin-7, which constitute the tight junctions of epithelial cells. As such, the destruction of intercellular junctions would assist the bacteria to invade the intestinal epithelial tissue, and thus pass through the intestinal barrier into deep sites in the human body, resulting in systemic infections.

The importance of the epithelial barrier in the prevention of bacterial invasion is also highlighted in another *in vitro* study in this special issue **(** Shrestha et al **).** Interestingly, the authors also factored in the interplay between the exposure to occupational hazards, such as organic dust (OD) & hydrogen sulfide (H_2_S), intactness of the epithelial barrier and bacterial invasion. Findings of this study demonstrated that repeated exposure to organic OD and H_2_S resulted in: increased oxidative stress, production of inflammatory cytokines, such as IL-6 and IL-1B, and most importantly in the loss of epithelial tight junction proteins and barrier integrity, which facilitated the invasion of *Klebsiella pneumoniae* in human airway epithelial cells and murine lung slices. These findings were associated with increased levels of Keap1 and decreased expression of Nrf2, two components that act together to tightly maintain the baseline and stimulated antioxidant responses. Interestingly, all these changes were rescued by pharmacologic activation of Nrf2, including bacterial invasion in the murine lung slice model. Mechanistic data from this study will be helpful in developing better therapeutic agents against exposure-induced loss of barrier function, and thus protect farm workers against various respiratory diseases.

Lastly, an opinion article by Srinivasan explored the potential effect of long COVID-19 on taste dysfunction and how bacteria harboring the tongue might contribute to this mechanism. The lingual epithelium is covered by “tongue film” which includes: exfoliated cells, residual saliva and the microbiota. In this context, taste sensitivity is modulated by the cellular density and the bacterial microbiota in the tongue film. Dysbiosis secondary to long viral infections, such as COVID-19, disrupts the commensal homeostasis and induces innate inflammatory responses, which results in an increased epithelial proliferation and exfoliation. As such, pressure to replace the exfoliated taste receptor cells by stem cells disrupts and alters the epithelial homeostasis, which in turn affects the taste perception.

Taking all the articles presented in this Research Topic together, invasion of pathogenic bacteria and bacterial dysbiosis have serious consequences entailed in the development of serious localized or systemic infections and in the impairment of important body functions, such as taste. Maintaining the epithelial barrier in different body organs plays a key role in protection against bacterial invasion. However, since bacteria can cause destruction of tight junctions in the epithelial barrier, further research will be needed on how to combat the bacterial mechanism of barrier destruction.

## Author Contributions

All authors have contributed equally to the editorial statement and approved the submitted version.

## Conflict of Interest

The authors declare that the research was conducted in the absence of any commercial or financial relationships that could be construed as a potential conflict of interest.

## Publisher’s Note

All claims expressed in this article are solely those of the authors and do not necessarily represent those of their affiliated organizations, or those of the publisher, the editors and the reviewers. Any product that may be evaluated in this article, or claim that may be made by its manufacturer, is not guaranteed or endorsed by the publisher.

